# Structural characterization of methylation-independent PP2A assembly guides alphafold2Multimer prediction of family-wide PP2A complexes

**DOI:** 10.1016/j.jbc.2024.107268

**Published:** 2024-04-04

**Authors:** Franziska Wachter, Radosław P. Nowak, Scott Ficarro, Jarrod Marto, Eric S. Fischer

**Affiliations:** 1 Department of Pediatric Oncology, Dana-Farber Cancer Institute, Boston, Massachusetts, USA; 2 Department of Cancer Biology, Dana-Farber Cancer Institute, Boston, Massachusetts, USA; 3 Department of Biological Chemistry and Molecular Pharmacology, Harvard Medical School, Boston, Massachusetts, USA

**Keywords:** X-ray crystallography, phosphatase, protein phosphatase 2 (PP2A), protein serine/threonine phosphatase (PSP), protein methylation

## Abstract

Dysregulation of phosphorylation-dependent signaling is a hallmark of tumorigenesis. Protein phosphatase 2 (PP2A) is an essential regulator of cell growth. One scaffold subunit (A) binds to a catalytic subunit (C) to form a core AC heterodimer, which together with one of many regulatory (B) subunits forms the active trimeric enzyme. The combinatorial number of distinct PP2A complexes is large, which results in diverse substrate specificity and subcellular localization. The detailed mechanism of PP2A assembly and regulation remains elusive and reports about an important role of methylation of the carboxy terminus of PP2A C are conflicting. A better understanding of the molecular underpinnings of PP2A assembly and regulation is critical to dissecting PP2A function in physiology and disease. Here, we combined biochemical reconstitution, mass spectrometry, X-ray crystallography, and functional assays to characterize the assembly of trimeric PP2A. *In vitro* studies demonstrated that methylation of the carboxy-terminus of PP2A C was dispensable for PP2A assembly *in vitro*. To corroborate these findings, we determined the X-ray crystal structure of the unmethylated PP2A Aα-B56ε-Cα trimer complex to 3.1 Å resolution. The experimental structure superimposed well with an Alphafold2Multimer prediction of the PP2A trimer. We then predicted models of all canonical PP2A complexes providing a framework for structural analysis of PP2A. In conclusion, methylation was dispensable for trimeric PP2A assembly and integrative structural biology studies of PP2A offered predictive models for all canonical PP2A complexes.

The modification of specific amino acid sidechains such as serine, threonine, or tyrosine with a phosphate group, phosphorylation, is a tightly regulated process. The dysregulation of phosphorylation-dependent signaling is a hallmark of leukemogenesis (1, 2) and other disease such as cancer or neurodegenerative disease (3). Kinases and phosphatases regulate phosphorylation by either adding or removing a phosphate group from proteins, respectively. Kinases are frequently mutated in disease, such as activating mutations in oncogenic kinases (4, 5, 6, 7). This led to the development of selective kinase inhibitors that are now a mainstay of targeted cancer therapies such as in the case of Imatinib (chronic myelogenous leukemia) or Osimertinib (lung cancer) (8, 9). Kinase inhibition also plays a crucial role in several previously hard-to-treat pediatric cancers, including metastatic ALK fusion-driven inflammatory myofibroblastic tumors (5). Conversely, it has been hypothesized that an orthogonal way to target aberrant phosphorylation pathways is to inhibit or activate phosphatases, with a first example targeting PTPN2 entering early clinical development (10).

The serine/threonine phosphatase PP2A represents a particularly important example as it serves tumor suppressive roles and unlike other tumor suppressors, inactivation commonly does not involve mutations or deletions, but rather inhibition or dysregulation of assembly. This, in principle, suggests that activation could be achieved by small molecules, but a lack of a clear understanding of the inactivating processes and overall assembly principles presents obstacles.

PP2A is an essential serine/threonine phosphatase that regulates various cellular processes, including cell growth, division, and differentiation. PP2A is functionally impaired in cancer through suppression of individual subunits and up-regulation of endogenous repressors (11). PP2A is a modular multi-subunit enzyme: One scaffold subunit (A) binds to a catalytic subunit (C) to form a core AC heterodimer, which together with one of many regulatory (B) subunits forms the active trimeric enzyme. The A- and C- subunits have two isoforms each. The 16 regulatory (B) subunits are classified into four distinct subfamilies with multiple isoforms each not comprising significant homology between subfamilies. The combinatorial assembly of these subunits generates a wide range of PP2A complexes, resulting in diverse substrate specificity and subcellular localization (12, 13, 14, 15). Historically phosphatases were deemed undruggable due to concerns that the highly conserved active site makes it difficult to achieve selectivity (16). Allosteric modulators could in principle achieve selectivity, but a substantial structural understanding of PP2A is needed for the development of such novel therapeutics.

The precise regulation and assembly of PP2A remains still a subject of active research, and conflicting findings have been reported regarding the role of carboxy-terminal PP2A C-subunit methylation (17, 18, 19, 20, 21, 22, 23, 24, 25, 26). Gaining insight into the molecular control of PP2A activity is crucial to facilitating the development of allosteric phosphatase activation as a novel therapeutic approach. Here, we provide a framework for structural and mechanistic studies of PP2A and clarify the role of the carboxy-terminal methylation of the PP2A C-subunit utilizing a combination of biochemical reconstitution, X-ray crystallography, and computational modeling.

## Results

### PP2A Aα-B55α-Cα trimer and PP2A Aα-B56ε-Cα trimer form spontaneously *in vitro* in an unmethylated form

**Figure 1:**
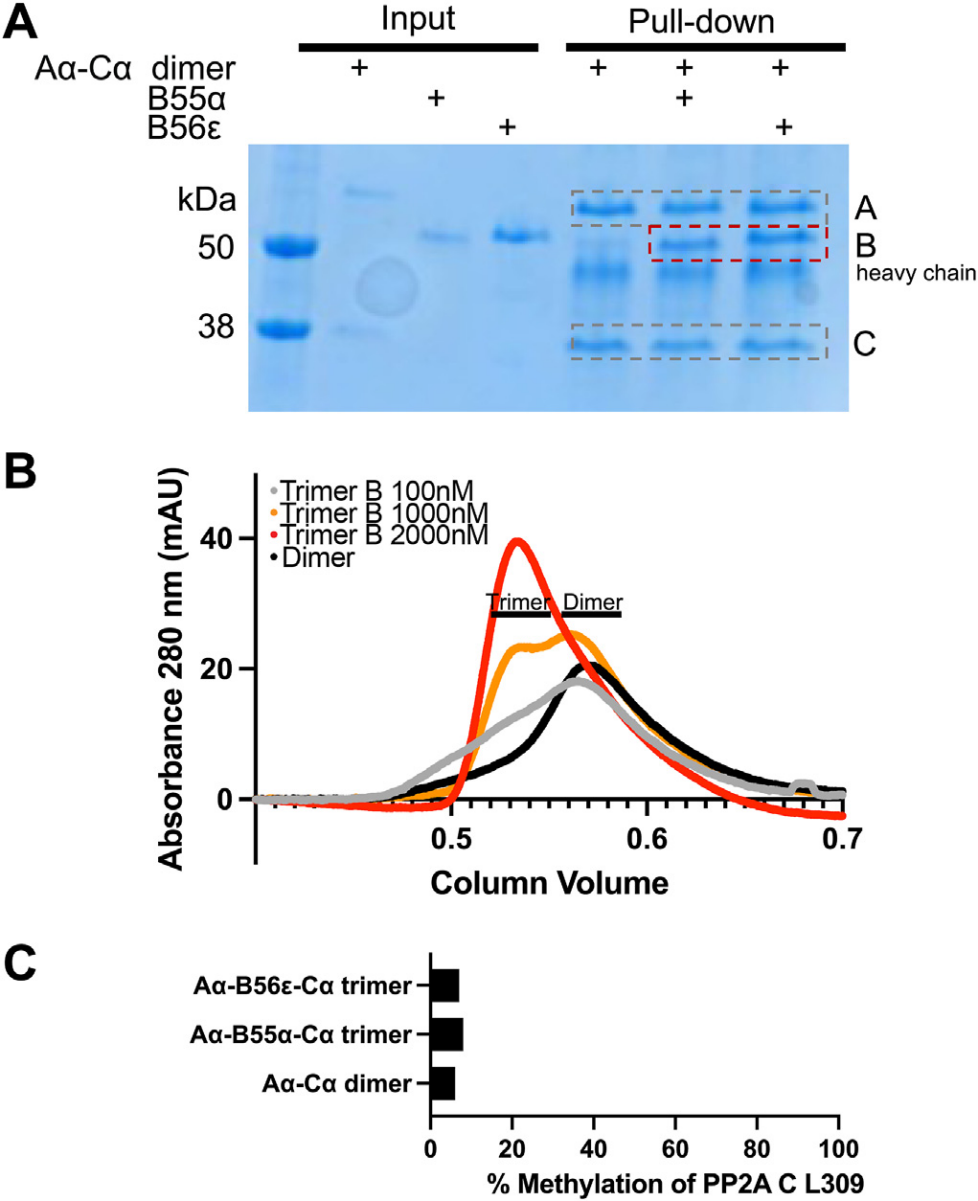
**PP2A Aα-B55α-Cα trimer and PP2A Aα-B56ε-Cα trimer form spontaneously *in vitro* in an unmethylated form.***A*, Flag pulldown visualizes PP2A complex formation. *B*, SEC demonstrates elution of PP2A Aα-B56ε-Cα as a trimer and confirms complex assembly, complex assembly was dependent on B56ε-subunit concentration. *C*, methylation status of PP2A was assessed by mass spectrometry.

During the purification of PP2A Aα-B55α-Cα trimer and PP2A Aα-B56ε-Cα trimer complexes, we noticed that co-expression in Hi-5 insect cells was sufficient for stable complex formation. Since complete site-specific methylation of the C-subunit is unlikely to occur in insect cells, this observation prompted us to further investigate the assembly of the complexes. Flag pull-down assays provided additional evidence that PP2A can spontaneously form *in vitro*: A Flag-tagged version of the PP2A A-subunit was expressed and purified, along with orthogonally tagged or untagged variants of other subunits. When mixed *in vitro*, the catalytic and regulatory subunit of PP2A naturally came together to create a functional enzyme (Fig. 1*A*). This spontaneous formation suggested that no additional components were necessary and showed an intricate self-assembly capacity of PP2A. Interestingly, both tested PP2A B-subunits formed spontaneously *in vitro* (Fig. 1*A*). The purity and stability of recombinant PP2A was confirmed (Figs. S1 and S2). We next performed analytical size exclusion chromatography (SEC) to investigate the formation of trimeric PP2A. Since SEC can separate PP2A A/B/C trimer from the A/C dimer and individual subunits, it provides an orthogonal confirmation of complex formation (27). SEC was performed on purified PP2A Aα-Cα dimer, or PP2A Aα-Cα dimer mixed with PP2A B56ε regulatory subunit at increasing concentration. Concentration-dependent assembly of functional trimers was observed (Fig. 1*B*). We confirmed that the proteins purified from insect cells are only partially methylated at the C-subunit carboxy-terminus (6–8% as quantified by mass spectrometry) and that this methylation was insufficient to explain the observed stoichiometric assembly (Fig. 1*C*, Table S2).

### Mass spectrometry-based analysis of PP2A C methylation

Methylation of the PP2A C-subunit has been broadly described to control complex assembly of PP2A (19, 28, 29), however, contradicting reports also exist (25, 26). Methylation of PP2A C is typically identified using antibodies developed to recognize the specific methyl mark on the carboxy group of leucine 309 of PP2A C (19). It is well established that antibodies specifically antibodies recognizing a modified species, require extensive validation and are not inherently quantitative. Antibodies utilized to identify PP2A C have demonstrated varying affinities for methylated and demethylated PP2A C (17, 21, 30). To overcome these limitations and accurately assess the methylation state of our purified, recombinant protein we utilized mass spectrometry as a readout. We showed that PP2A Aα-Cα heterodimer, PP2A Aα-B55α-Cα trimer, and PP2A Aα-B56ε-Cα trimer were not substantially methylated using mass spectrometry (Fig. 1*C*). Additionally, recombinant PP2A expressed in mammalian cells was not methylated (Table S2). We confirmed that the purified proteins from insect cells are only partially methylated and that this methylation was insufficient to explain stochiometric assembly (Fig. 1*C*).

### Carboxy-terminal PP2A C methylation does not affect phosphatase activity

**Figure 2:**
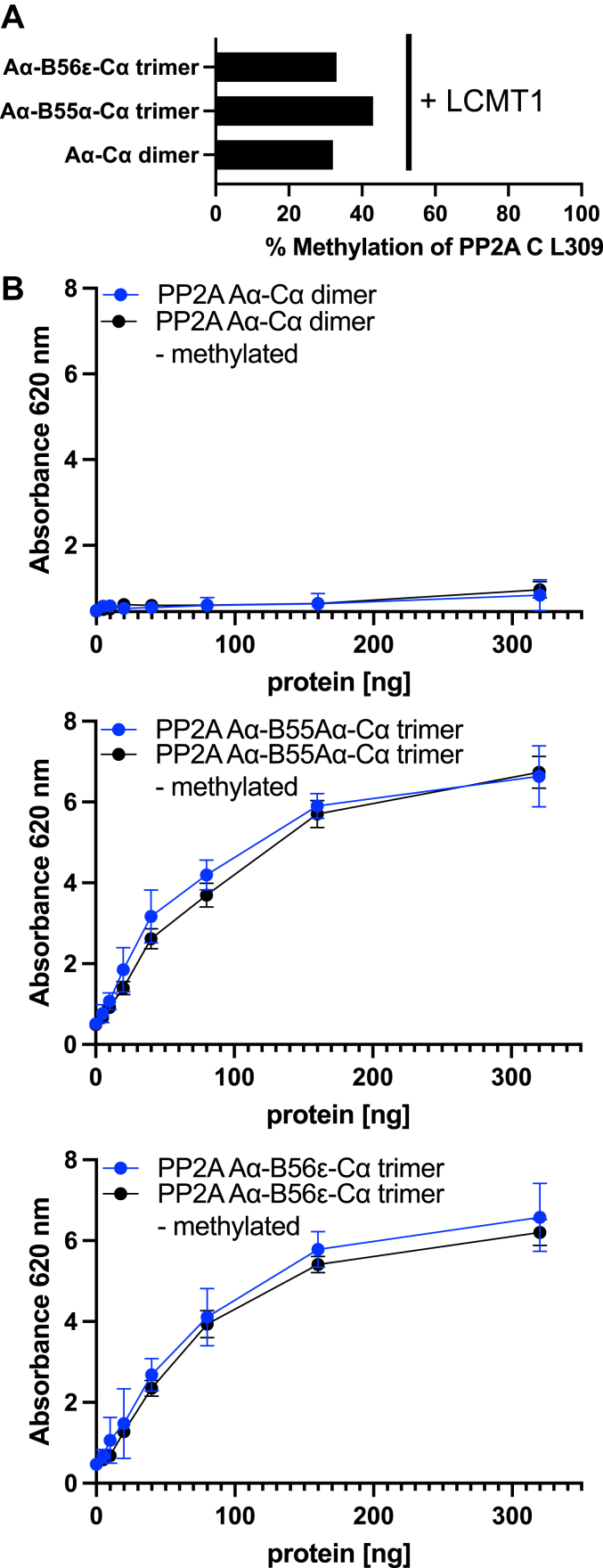
**Carboxy-terminal PP2A C methylation does not affect phosphatase activity.***A*, Recombinant demethylated PP2A is *B*, enzymatically active at a similar level as LCMT-1 methylated PP2A.

With an established method in place to accurately quantify the methylation state of PP2A Cα, we next sought to investigate whether methylation while not affecting assembly, may affect phosphatase activity. Phosphatase activity of PP2A was assessed through calorimetric assays employing a chemically synthesized phosphopeptide, RRA(pT)VA, as a substrate and followed by detection of free orthophosphate released by phosphatase activity using malachite green reagent (31). To compare methylated and non-methylated complexes, we subjected purified PP2A to methylation with the canonical methyl transferase LCMT1 and confirmed methylation of the carboxy group of leucine 309 of PP2A C by mass spectrometry to be 30 to 40% (Fig. 2*A*). Next, we subjected unmethylated and methylated PP2A Aα-B55α-Cα trimer and PP2A Aα-B56ε-Cα trimer to the phosphatase assay and found that phosphatase activity is similar between methylated and unmethylated PP2A (Fig. 2*B*). PP2A AC heterodimer did not exhibit substantial phosphatase activity. Similar results were obtained when assaying phosphatase activity with the generic p-nitrophenyl phosphate substrate (Fig. S3). These findings additionally confirm that the formation of the PP2A Aα-B55α-Cα trimer and the PP2A Aα-B56ε-Cα trimer occurs spontaneously, irrespective of methylation. Moreover, methylation does not appear to govern PP2A activity towards a peptide substrate.

### X-ray crystal structure of the PP2A Aα-B56ε-Cα trimer complex to 3.1 Å resolution with the unmethylated carboxy group of leucine 309

**Figure 3:**
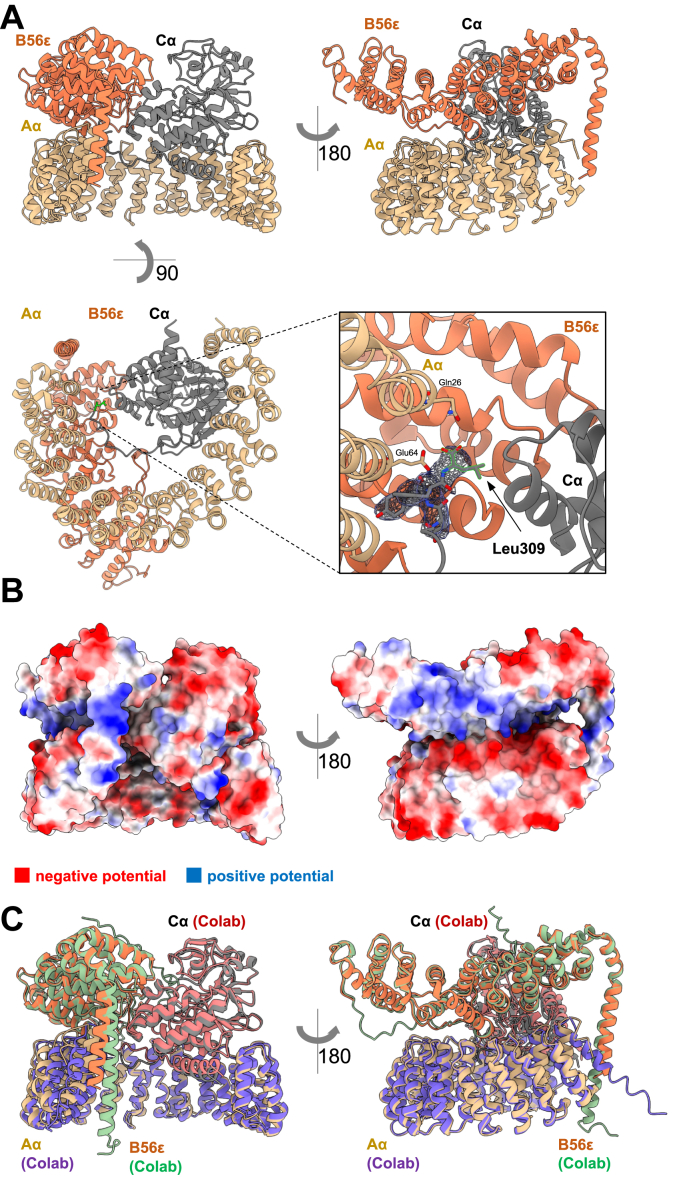
**X-ray crystal structure of PP2A Aα-B56ε-Cα complex at 3.1 Å resolution with the unmethylated carboxy group of leucine 309.***A*, overall structure of the PP2A Aα-B56ε-Cα complex. The B-subunit (B56ε, PPP2R5E) forms an arc-like protein consisting of 20 alpha helices and the B-subunit makes extensive contacts with the A-subunit with a notable over 25 amino acid long alpha helix of the B-subunit connecting to the N-terminus of the A-subunit. The B-subunit forms an interface with the C-subunit. The C-subunit C-terminal tail ending with the leucine 309 is clearly visible and wedges itself at the interface between the three subunits. *B*, calculated coulombic electrostatic surface potential indicating high charge complementarity between subunits. *C*, overlay of the X-ray crystal structure of PP2A Aα-B56ε-Cα complex with the Alphafold2Multimer (AF2M) prediction generated in ColabFold.

To corroborate the above findings, we determined the X-ray crystal structure of the PP2A Aα-B56ε-Cα trimer complex at 3.1 Å resolution with the unmethylated carboxy-group of leucine 309 (Fig. 3). Interestingly our X-ray crystal structure overlayed well with an Alphafold2Multimer (AF2M) model of the PP2A trimer (Fig. 3*C*). The Aα and Cα subunits formed a previously described complex (32). The B56ε B-subunit forms an arc-like protein consisting of 20 alpha helices. The B-subunit makes extensive contacts with the A-subunit (interface area of 1384 Å^2^), an interaction which is largely based on the charge complementarity of a negatively charged A-subunit and a positively charged B-subunit (Fig. 3*B*). Notably, the structure of the C-terminus of the B56ε-subunit includes an over 25 amino acid long alpha helix stabilized by the connection to the N-terminus of the A-subunit. The B-subunit interacts with the C-subunit with a large 1294 Å^2^ interface. The carboxy-terminal tail of PP2A C ending with the leucine 309 is clearly visible and wedges itself at the intersection between the three subunits (Fig. 3*A*). Given the recent successes with the prediction of complexes with novel multi-subunit structure prediction methods, we asked whether AF2M would be able to recapitulate the experimentally observed ternary complex. Interestingly not only did our X-ray crystal structure superimpose well with an AF2M model of the PP2A trimer (RMSD: 1.047 Å), but also all the key features of the interface including the >25 amino acid long B-subunit helix were predicted (Fig. 3*C*).

### Prediction of all canonical PP2A complexes provides a framework for PP2A assembly

**Figure 4:**
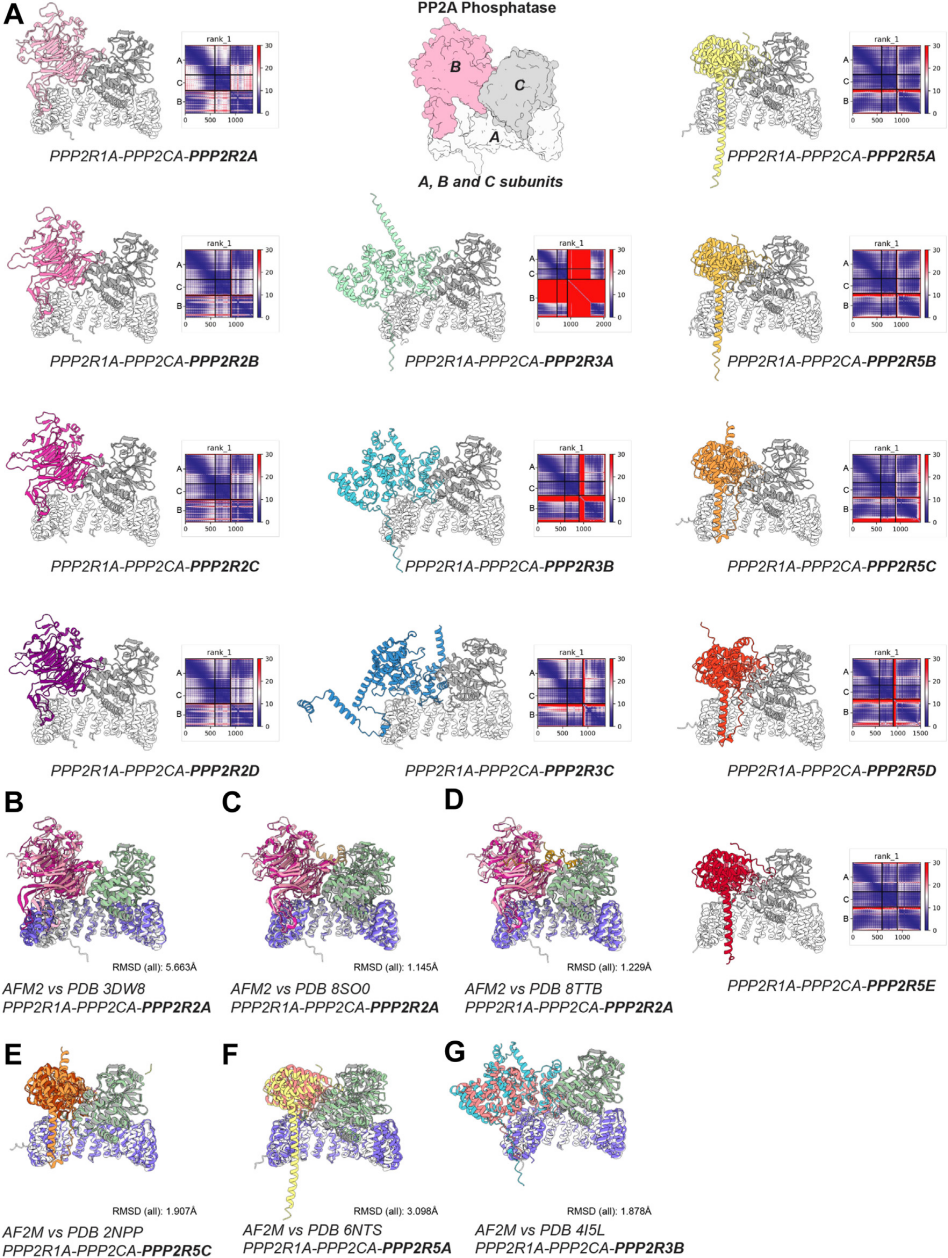
**Alphafold2Multimer (AF2M) prediction of PP2A complexes.***A*, AF2M predicted the PP2A Aα-B56ε-Cα trimer and found striking alignment when superimposed on our experimental structure. Generated models of the entire PP2A family provide a framework of PP2A assembly. The predicted aligned error (PAE) plots are shown in heatmaps, where X and Y axis correspond to residues within each chain (*A*–*C*). The PAE is a distance error between two residues measured in Angstroms in a range 0 to 30. It describes certainty in position of residue x when predicted and true structures are aligned on residue y. High off-diagonal PAE, say between chains A and B, indicates high confidence in predicted complex. The PAE plot is shown for all residues in a heatmap color coded low (*blue*) to high (*red*). *B*, alignment of AF2M prediction of PPP2R1A-PPP2CA-PPP2R2A complex with X-ray crystal structure PDB: 3DW8. The PPP2R2A of X-ray structure is shown in deep *pink* and AF2M coloring corresponds to *panel A*. *C*, as in *A* but AF2M prediction is aligned with cryo-EM structure PDB: 8SO0. *D*, as in *A* but AF2M prediction is aligned with cryo-EM structure PDB: 8TTB. *E*, alignment of AF2M prediction of PPP2R1A-PPP2CA-PPP2R5C with X-ray crystal structure PDB: 2NPP. The PPP2R5C of X-ray structure is shown in *light orange* and AF2M coloring corresponds to *panel A*. *F*, alignment of AF2M prediction of PPP2R1A-PPP2CA-PPP2R5A with cryo-EM structure PDB: 6NTS. The PPP2R5A of X-ray structure is shown in *yellow* and AF2M coloring corresponds to *panel A*. *G*, alignment of AF2M prediction of PPP2R1A-PPP2CA-PPP2R3A with X-ray crystal structure PDB: 4I5L. The PPP2R3A of the X-ray structure is shown in *light pink* and AF2M coloring corresponds to *panel A*.

Building on the successful prediction of the experimental structure of the PP2A Aα-B56ε-Cα trimer, we next generated models of the entire PP2A family to provide a framework for PP2A assembly (Fig. 4*A*). We performed our predictions by co-folding 1:1:1 A, B and C subunits of PP2A. AF2M was not able to predict PP2A complexes with Striatin (B subunit) or PTPA. Striatin is a B-subunit that is largely unstructured. In the experimental setting, Striatin also did not form a trimeric complex with PP2A suggestive of a missing partner within this complex. These results are consistent with the recently published cryo-EM structure of the Striatin-interacting phosphatase and kinase (STRIPAK) complex, with four copies of STRN3 and one copy of each of the PP2A A-C heterodimer (33). To assess the accuracy of the prediction further, we overlayed predicted complexes with available structures (Fig. 4, *B*–*D*). Overall, PP2A predictions were consistent with available structures. Notably, the alignment of the experimental and predicted structure visualized an unusually long alpha helix of B56ε in the prediction for the whole B’ (PR61, PPP2R5) family. AF2M models are colored by pLDDT score (blue – high, red – low). The predicted aligned error (PAE) plots indicating high confidence in complex prediction are provided in the supplementary data (Fig. S4).

## Discussion

PP2A is a central mediator of key cellular and physiologic functions including oncogenic signaling pathways. Hence, altering PP2A assembly holds considerable promise for therapeutic intervention (34). However, complex multimeric structures with numerous isoforms make mechanistic studies of PP2A challenging. Here, we demonstrated that PP2A formed spontaneously *in vitro* and methylation of the carboxyterminal leucine of the C-subunit was dispensable for its formation. We then used structural information obtained by X-ray crystallography and AF2M modeling to provide a framework for assembly as a basis for further mechanistic studies. Through thorough in-vitro studies, we further revealed that PP2A can spontaneously form and exhibit enzymatic activity even in the absence of leucine 309 carboxy-terminal methylation. The requirement for C-terminal methylation did not apply to the formation of PP2A Aα-B55α-Cα and PP2A Aα-B56ε-Cα trimer. Importantly our study suggests that previous studies that use leucine 309-specific antibodies may need to be interpreted with caution (17).

While methylation-independent activation was established for two specific phosphatases, its applicability to other phosphatases remains to be explored. Methylation may still play a regulatory role for specific PP2A assemblies. Our studies, together with previous work highlight the complexity of the assembly of multi-subunit enzymes and their regulation. The interplay between cellular inhibitors and activators remains intricate, with specificity for distinct trimeric assemblies awaiting firm establishment. Additional regulatory mechanisms likely exist, although they may not be universally applicable to all B-subunits. For example, Kaynak *et al.* demonstrated the unique ability of the PP2A A-subunit architecture to adapt to functional inputs through changes in conformation in the response to mechanical stress (35). While our work provides another small piece of the puzzle, a comprehensive understanding of PP2A regulation remains elusive and requires subsequent studies including thorough *in vitro* reconstitutions and structural characterization.

Given the modularity and structural complexity of the PP2A family, we used AF2M predictions to obtain additional models for making inferences that can be tested experimentally. Comparing overlayed AF2M models with previously published structures revealed a remarkable alignment, strengthening confidence in the accuracy of the generated models. For example, AF2M correctly predicted an unusually long alpha helix of the B-subunit, validated by X-ray crystallography with B56ε, and featuring in all B’ (PR61, PPP2R5) family members. Although experimental validation is necessary, AF2M predictions suggest a significant similarity between B-subunits within the B (PR55, PPP2R2) and B’ (PR61, PPP2R5) families, with the greatest diversity observed in the B’’ (PPP2R3) family. The ability to obtain high-confidence models with AF2M provides an important toolset for the functional dissection of the PP2A family. For example, the accurate prediction of protein-protein interfaces (PPI), may enable researchers to rationalize loss of function mutations inactivating specific B-subunit-containing complexes without affecting PP2A activity. Alternatively, gain of function mutations could be designed to create constitutively active trimeric enzymes. While not all N- or C-terminal regions of predicted protein structures were visible by X-ray crystallography or cryo-EM, it is conceivable that these regions play key regulatory roles. Post-translational modifications in these regions may contribute to the regulation of assembly or activity. Our and others experimental structures confirm that AF2M is capable of generating high-confidence models of the entire PP2A family except for the PP2A Striatin complex, which also did not form a complex with A/B alone in our experimental setting. Our study thus provides additional insights into trimeric PP2A modulation. The structural and functional data in combination with AF2M predictions can serve as a basis for new hypotheses and may aid in the development of mechanism-based therapeutics.

## Experimental procedures

### Protein purification

Recombinant PP2A dimer/trimer (Flag-tagged A-subunit, non-tagged B-subunit, and strep-tagged C-subunit) or individual strep-tagged B-subunits were expressed in insect cells, and purified by strep-tactin affinity purification, anion exchange chromatography and size exclusion chromatography (SEC). Extensive quality control, including liquid chromatography–mass spectrometry (LC-MS), SDS-PAGE gel and fast protein liquid chromatography was performed. We validated protein stability with differential scanning fluorimetry (Figs. S1 and S2). Construct information and further experimental details can be found in the supplementary data.

### Complex formation of PP2A

Pull-down assays and size exclusion chromatography characterized complex formation. Recombinant PP2A AC dimer and individual B-subunits were incubated at 1 μM final concentration for 15 min at room temperature. Flag beads (sigma) were added and incubated for 4 h at 4 °C rotating. The supernatant was removed and beads were washed 5 times with RIPA buffer. Protein was eluted at 95 °C for 10 min. Coomassie brilliant blue R-250 was used to stain protein samples after electrophoretic separation in a 4 to 20% polyacrylamide gel.

### Functional studies of PP2A

Functional evaluations of PP2A were conducted through calorimetric assays employing both, a chemically synthesized phosphopeptide, RRA(pT)VA, and p-nitrophenyl phosphate as a substrate in a 96-well configuration, employing colorimetric detection facilitated by a plate reader operating at 620 nm and 405 nm, respectively. This approach allowed for robust characterization of the PP2A function (31). Further details are outlined in the supplementary data.

### Mass spectrometry to characterize methylation of PP2A

The degree of methylation of the carboxy group of PP2A C leucine 309 was confirmed by mass spectrometry. PP2A protein complex (1 μg) was diluted with 100 mM ammonium bicarbonate and reduced with 10 mM dithiothreitol for 30 min at 56 °C. After cooling to room temperature, cysteines were alkylated with 22.5 mM iodoacetamide for 30 min protected from light. Glu-C (100 ng) was added, and the solution was incubated overnight at 37 °C. Peptides were acidified with 10% formic acid, desalted by C18, and dried by vacuum centrifugation. Peptides were reconstituted in 3% MeCN/0.1% formic acid and analyzed by nano-LC/MS using a Nanoacquity UPLC system (Waters) coupled to a QExactive HF mass spectrometer (ThermoFisher Scientific). Peptides were injected onto a self-packed precolumn (4 cm Symmetry C18, Waters) at a flow rate of 3 μl/min and resolved on an analytical column with integrated emitter tip (0.5 m Monitor C18, Orochem, 30 μm I.D., flow rate ∼30 nl/min (PMID: 19331382)) using an LC gradient (2–35% B in 60 min, A = 0.1% formic acid in water, B = 0.1% formic acid in acetonitrile). The mass spectrometer was operated in data-dependent mode and the 10 most abundant ions were subjected to MS/MS (NCE = 30%, resolution = 15k, max fill time = 100 ms, target = 1E5). MS data was converted to.mgf using multiplierz scripts (36) and peptides were identified using Mascot version 2.6.1 searching a custom database of laboratory proteins containing PP2A sequences. Precursor and product ion tolerances were 10 ppm and 0.025 Da, respectively. Search parameters specified fixed carbamidomethylation of cysteine, variable methionine oxidation, and variable C-terminal methyl ester modification. Peak areas of peptides were calculated using mzStudio (37).% methylation was calculated as (methyl ester modified L309 signal)/(unmodified L309 signal + methyl ester modified L309 signal) after normalizing against additional PP2A Glu-C peptides.

### Crystallization and data collection

For crystallization of the PP2A Aα-B56ε-Cα ternary complex, we have set up crystallization plates in three sub-well plates (Intelli, Art Robbins) by vapor diffusion using NT8 (Formulatrix) with 150 nl drops and 2:1, 1:1 or 1:2 protein to precipitant ratio. Crystal plates were set up at 4 °C and 20 °C and images were acquired using RockImager 1000 (Formulatrix). Plate-like crystals initially appeared in TOP96 screen wells B2 and D8 and were further optimized in a solution containing Bis-Tris buffer and PEK20k precipitant. The final screen of the collected crystal was 23% PEG20k and 0.1 M Bis-Tris (pH 5.77). Crystals were cryo-protected in a reservoir solution supplemented with 20% glycerol containing 1 mM of 22,980, a PPZ analog that was not identified in the crystal structure, and flash–cooled in liquid nitrogen. Diffraction data were collected at the APS Chicago (beamline 24-ID-C) with a Pilatus 6M-F detector at a temperature of 100 K. Data were processed using the RAPD pipeline (APS Chicago) which utilizes XDS (38) and CCP4 suite (39) programs to scale the data. Data processing statistics, refinement statistics, and model quality parameters are provided in Table S1.

### Structure determination and model building

The PP2A Aα-B56ε-Cα ternary complex crystallized in space group P1 with two complexes in the unit cell. PHASER (40) was used to determine the structures by molecular replacement using a crystallographic model of PP2A Aα-B56α-Cα based on a crystal structure PDB: 2NPP. The initial model was iteratively improved with COOT (41) and refined using PHENIX.REFINE (42) and compared to the prediction generated in ColabFold (43). Protein geometry analysis revealed 0.15% Ramachandran outliers, with 94.97% residues in favored regions and 4.88% residues in allowed regions. Figures were generated with PyMOL (The PyMOL Molecular Graphics System, Version 2.5.5 Schrödinger, LLC) and model quality was assessed with MOLPROBITY (44). The structure was analyzed by PISA v1.52 (44) available at the PDBe to calculate the interface areas.

## Data availability

Obtained protein structure of the PP2A Aα-B56ε-Cα is available at the RCSB Protein Data Bank under the accession number: 8UWB. The generated AF2M models is available at Zenodo (DOI: 10.5281/zenodo.10802473) for download. The data that support the findings of this study are available from the corresponding author upon reasonable request.

## Supporting information

This article contains supporting information (43, 45, 46, 47, 48).

## Conflict of interest

The authors declare the following financial interests/personal relationships which may be considered as potential competing interests:

E. S. F is a founder, scientific advisory board (SAB) member, and equity holder of Civetta Therapeutics, Proximity Therapeutics, and Neomorph, Inc (also board of directors). He is a SAB member and equity holder for Avilar Therapeutics, Ajax Therapeutics and Photys Therapeutics, an equity holder in Light Horse Therapeutics and a consultant to Novartis, Sanofi, EcoR1 Capital, Odyssey, and Deerfield. The Fischer lab receives or has received research funding from Deerfield, Novartis, Ajax, Interline and Astellas.
