# Supplementary material for: Structural characterization of methylation-independent PP2A assembly guides alphafold2Multimer prediction of family-wide PP2A complexes
Source: J Biol Chem. 2024 Apr 4;300(5):107268. doi: 10.1016/j.jbc.2024.107268 (PMC11087950; doi:10.1016/j.jbc.2024.107268)
Supplement: Supporting Information [file mmc1.docx]

**Supporting Information**

**Structural characterization of methylation-independent PP2A assembly guides Alphafold2Multimer prediction of family-wide PP2A complexes**

Franziska Wachter^1,2,3^, Radosław P. Nowak^2,3^, Scott Ficarro^2^, Jarrod Marto^2^, Eric S. Fischer^2,3^

^1^ Department of Pediatric Oncology, Dana-Farber Cancer Institute, Boston, MA, USA

^2^ Department of Cancer Biology, Dana-Farber Cancer Institute, Boston, MA, USA

^3^ Department of Biological Chemistry and Molecular Pharmacology, Harvard Medical School, Boston, MA, USA

*Correspondence to: Eric S. Fischer (Eric_Fischer@dfci.harvard.edu).

Contents Page

Figure S1 S3

Figure S2 S4

Figure S3 S5

Figure S4 S6

Supplementary Table 1 S7

Supplementary Table 2 S8

Constructs and protein purification S9

Differential scanning fluorimetry S10

In vitro methylation of purified PP2A protein S10

Phosphatase activity assay S11

ColabFold model preparation S11

References S14

**Figure S1.** Coomassie gel, anion exchange and size exclusion demonstrate purity.


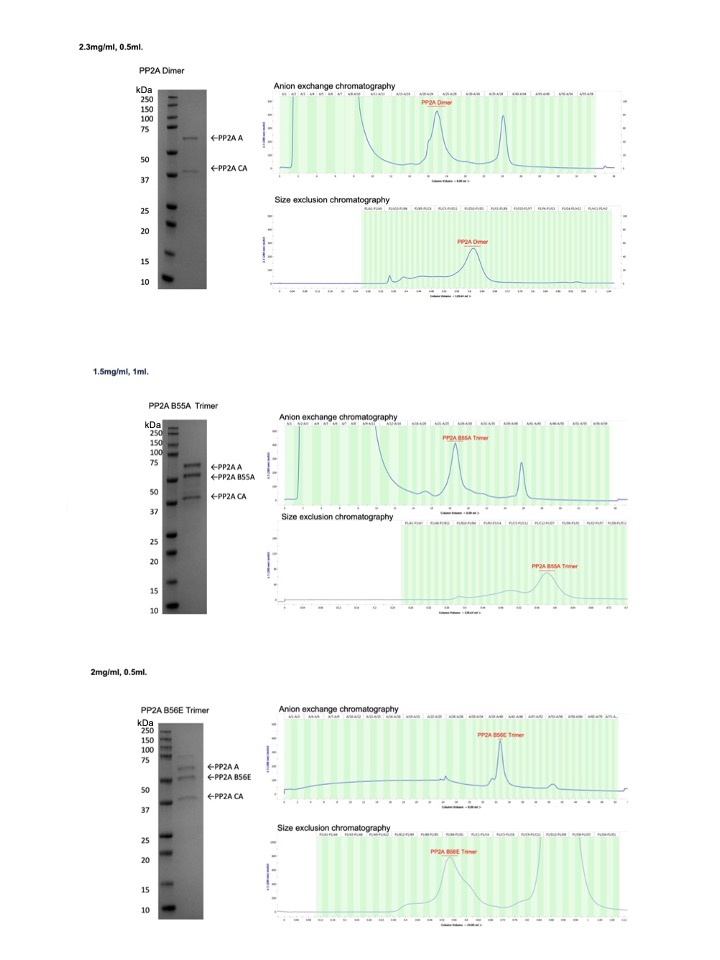

**Figure S2.** Differential scanning fluorometry demonstrates temperature stability of PP2A.

**Figure S3.** Phosphatase activity of methylated and non-methylated PP2A utilizing generic p-nitrophenyl phosphate substrate. Phosphatase activity was measured in triplicate and normalized to the absorbance of the maximum phosphatase activity (determined by using commercial bovine alkaline phosphatase (20mcg/ml final concentration, Sigma). Phosphatase activity was expressed in percentage activity.


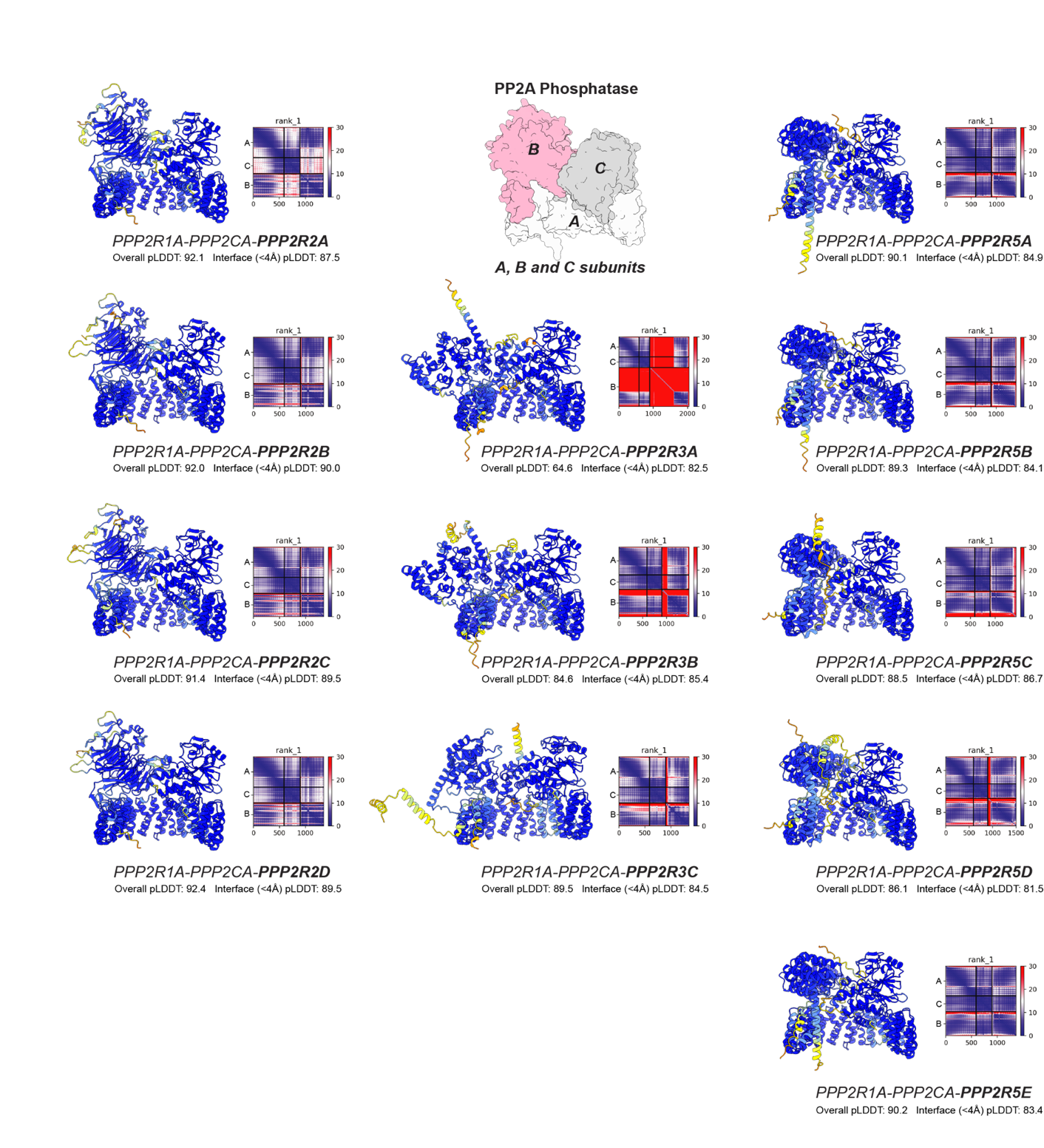


Figure S4. Corresponding to Figure 4. ColabFold models colored by predicted local distance difference test (pLDDT) score, a per-residue confidence score (blue – high, red – low) and their Predicted Aligned Error (PAE) plots indicating high confidence in complex prediction. The PAE is a distance error between two residues measured in Angstroms in a range 0-30. It describes certainty in position of residue x when predicted and true structures are aligned on residue y. High off-diagonal PAE, say between chains A and B, indicates high confidence in predicted complex. The PAE plot is shown for all residues in a heatmap color coded low (blue) to high (red). The overall pLDDT is calculated using all atoms in the structure, while Interface pLDDT is limited to atoms present at the interface between chains.

**Table S1. Data collection and refinement statistics.**

|  | **PDB ID 8UWB  PPP2R1A-PPP2R5E-PPP2CA complex** |
| --- | --- |
| **Wavelength** |  |
| **Resolution range (**Å) | 128.4 - 3.15 (3.263 - 3.15) |
| **Space group** | P 1 |
| **Unit cell (**Å, Å, Å, °, °, °) | 83.839 92.597 134.657  83.7723 72.4613 73.46 |
| **Total reflections** | 461927 (41979) |
| **Unique reflections** | 62227 (5795) |
| **Multiplicity** | 7.4 (6.8) |
| **Completeness (%)** | 96.42 (90.61) |
| **Mean I/sigma(I)** | 9.16 (0.82) |
| **Wilson B-factor** | 107.65 |
| **R-merge** | 0.154 (2.011) |
| **R-meas** | 0.1655 (2.177) |
| **R-pim** | 0.06023 (0.821) |
| **CC1/2** | 0.998 (0.397) |
| **CC*** | 0.999 (0.754) |
| **Reflections used in refinement** | 61723 (5793) |
| **Reflections used for R-free** | 3053 (263) |
| **R-work** | 0.2235 (0.4164) |
| **R-free** | 0.2642 (0.4645) |
| **CC(work)** | 0.955 (0.584) |
| **CC(free)** | 0.924 (0.485) |
| **Number of non-hydrogen atoms** | 20863 |
| **macromolecules** | 20859 |
| **ligands** | 4 |
| **solvent** | 0 |
| **Protein residues** | 2597 |
| **RMS(bonds)** | 0.002 |
| **RMS(angles)** | 0.52 |
| **Ramachandran favored (%)** | 94.66 |
| **Ramachandran allowed (%)** | 5.03 |
| **Ramachandran outliers (%)** | 0.31 |
| **Rotamer outliers (%)** | 0.00 |
| **Clashscore** | 8.33 |
| **Average B-factor** | 117.02 |
| **macromolecules** | 117.02 |
| **ligands** | 116.39 |
| **Number of TLS groups** | 1 |

Statistics for the highest-resolution shell are shown in parentheses.

**Table S2. Methylation of PP2A by mass spectrometry.**

**Constructs and Protein Purification**

Plasmid: psPAX2 Trono lab, EPFL, France, Addgene plasmid #12260.

Plasmid: pAC8RedNK Abdulrahman et al., 2009.

Human cDNA constructs of PPP2R1A, PPP2CA and PPP2R5E (B56ε) were synthesized by Twist. Unique amino acid tags of Flag (PPP2R1A), StrepII (PPP2CA) and none or StrepII [PPP2R5E (B56ε), PPP2R2A (B55α)], were designed to the N-terminal ends of the constructs, and then subcloned into pAC-derived expression vectors (pAC8RedNK) (Abdulrahman et al., 2009). Two µg of each pAC8RedNK vector, together with 0.5 µg of linearized baculoviral DNA, were transiently transfected into Sf-9 cells using FuGene HD (Promega) and ESF 921 Insect Cell Culture medium (Expression Systems), according to the manufacturer’s recommendation. The supernatant was sequentially collected to obtain baculovirus encoding each subunit of PP2A. Collected viruses were then used to infect High Five cells for protein expression in SF-900 II SFM medium (Thermo Fisher Scientific). High Five cells expressing each subunit of human PP2A were resuspended in 50 mM Tris-HCl (pH 8.0), 200 mM NaCl, 2mM Tris-(2-carboxyethyl)phosphine (TCEP), 1mM phenylmethylsulfonyl fluoride (PMSF) and protease inhibitor cocktail (Sigma) and lysed by sonication. Following ultracentrifugation, the soluble fraction was passed over the appropriate affinity resin of anti-Flag M2 affinity gel (Sigma-Aldrich; #A2220) for Flag-tagged PPP2R1A, Strep-Tactin XT Superflow (IBA) for StrepII-tagged PPP2R5E (B56ε), StrepII-tagged PPP2R2A (B55α), then eluted with wash buffer [50 mM Tris-HCl (pH 8.0), 200 mM NaCl and 1 mM TCEP] supplemented with 150 ng/ml 3× Flag Peptide (Millipore; F4799), 50 mM D-biotin (IBA), respectively. Extracted proteins were electrophoresed using agarose gels and the purity of each protein was assessed with SuperBlue Ultra Coomassie stain (Protea Biosciences).

Amino acid sequences of the PP2A subunits purified in this work:

Flag-TEV-site-R1A (A-subunit):

MDYKDDDDKSAVDENLYFQGGGRMAAADGDDSLYPIAVLIDELRNEDVQLRLNSIKKLSTIALALGVERTRSELLPFLTDTIYDEDEVLLALAEQLGTFTTLVGGPEYVHCLLPPLESLATVEETVVRDKAVESLRAISHEHSPSDLEAHFVPLVKRLAGGDWFTSRTSACGLFSVCYPRVSSAVKAELRQYFRNLCSDDTPMVRRAAASKLGEFAKVLELDNVKSEIIPMFSNLASDEQDSVRLLAVEACVNIAQLLPQEDLEALVMPTLRQAAEDKSWRVRYMVADKFTELQKAVGPEITKTDLVPAFQNLMKDCEAEVRAAASHKVKEFCENLSADCRENVIMSQILPCIKELVSDANQHVKSALASVIMGLSPILGKDNTIEHLLPLFLAQLKDECPEVRLNIISNLDCVNEVIGIRQLSQSLLPAIVELAEDAKWRVRLAIIEYMPLLAGQLGVEFFDEKLNSLCMAWLVDHVYAIREAATSNLKKLVEKFGKEWAHATIIPKVLAMSGDPNYLHRMTTLFCINVLSEVCGQDITTKHMLPTVLRMAGDPVANVRFNVAKSLQKIGPILDNSTLQSEVKPILEKLTQDQDVDVKYFAQEALTVLSLA

R2A (B55α-subunit)

MAGAGGGNDIQWCFSQVKGAVDDDVAEADIISTVEFNHSGELLATGDKGGRVVIFQQEQENKIQSHSRGEYNVYSTFQSHEPEFDYLKSLEIEEKINKIRWLPQKNAAQFLLSTNDKTIKLWKISERDKRPEGYNLKEEDGRYRDPTTVTTLRVPVFRPMDLMVEASPRRIFANAHTYHINSISINSDYETYLSADDLRINLWHLEITDRSFNIVDIKPANMEELTEVITAAEFHPNSCNTFVYSSSKGTIRLCDMRASALCDRHSKLFEEPEDPSNRSFFSEIISSISDVKFSHSGRYMMTRDYLSVKIWDLNMENRPVETYQVHEYLRSKLCSLYENDCIFDKFECCWNGSDSVVMTGSYNNFFRMFDRNTKRDITLEASRENNKPRTVLKPRKVCASGKRKKDEISVDSLDFNKKILHTAWHPKENIIAVATTNNLYIFQDKVN

StrepII-TEV-site-R2A (StrepII-B55α-subunit)

MDWSHPQFEKSAVDENLYFQGGGRMAGAGGGNDIQWCFSQVKGAVDDDVAEADIISTVEFNHSGELLATGDKGGRVVIFQQEQENKIQSHSRGEYNVYSTFQSHEPEFDYLKSLEIEEKINKIRWLPQKNAAQFLLSTNDKTIKLWKISERDKRPEGYNLKEEDGRYRDPTTVTTLRVPVFRPMDLMVEASPRRIFANAHTYHINSISINSDYETYLSADDLRINLWHLEITDRSFNIVDIKPANMEELTEVITAAEFHPNSCNTFVYSSSKGTIRLCDMRASALCDRHSKLFEEPEDPSNRSFFSEIISSISDVKFSHSGRYMMTRDYLSVKIWDLNMENRPVETYQVHEYLRSKLCSLYENDCIFDKFECCWNGSDSVVMTGSYNNFFRMFDRNTKRDITLEASRENNKPRTVLKPRKVCASGKRKKDEISVDSLDFNKKILHTAWHPKENIIAVATTNNLYIFQDKVN

R5E (B56ε-subunit)

MSSAPTTPPSVDKVDGFSRKSVRKARQKRSQSSSQFRSQGKPIELTPLPLLKDVPSSEQPELFLKKLQQCCVIFDFMDTLSDLKMKEYKRSTLNELVDYITISRGCLTEQTYPEVVRMVSCNIFRTLPPSDSNEFDPEEDEPTLEASWPHLQLVYEFFIRFLESQEFQPSIAKKYIDQKFVLQLLELFDSEDPRERDYLKTVLHRIYGKFLGLRAFIRKQINNIFLRFVYETEHFNGVAELLEILGSIINGFALPLKAEHKQFLVKVLIPLHTVRSLSLFHAQLAYCIVQFLEKDPSLTEPVIRGLMKFWPKTCSQKEVMFLGELEEILDVIEPSQFVKIQEPLFKQIAKCVSSPHFQVAERALYYWNNEYIMSLIEENSNVILPIMFSSLYRISKEHWNPAIVALVYNVLKAFMEMNSTMFDELTATYKSDRQREKKKEKEREELWKKLEDLELKRGLRRDGIIPT

StrepII-TEV-site-R5E (StrepII-B56ε-subunit)

MDWSHPQFEKSAVDENLYFQGGGRMSSAPTTPPSVDKVDGFSRKSVRKARQKRSQSSSQFRSQGKPIELTPLPLLKDVPSSEQPELFLKKLQQCCVIFDFMDTLSDLKMKEYKRSTLNELVDYITISRGCLTEQTYPEVVRMVSCNIFRTLPPSDSNEFDPEEDEPTLEASWPHLQLVYEFFIRFLESQEFQPSIAKKYIDQKFVLQLLELFDSEDPRERDYLKTVLHRIYGKFLGLRAFIRKQINNIFLRFVYETEHFNGVAELLEILGSIINGFALPLKAEHKQFLVKVLIPLHTVRSLSLFHAQLAYCIVQFLEKDPSLTEPVIRGLMKFWPKTCSQKEVMFLGELEEILDVIEPSQFVKIQEPLFKQIAKCVSSPHFQVAERALYYWNNEYIMSLIEENSNVILPIMFSSLYRISKEHWNPAIVALVYNVLKAFMEMNSTMFDELTATYKSDRQREKKKEKEREELWKKLEDLELKRGLRRDGIIPT

StrepII-TEV-site-CA (C-subunit)

MDWSHPQFEKSAVDENLYFQGGGRMDEKVFTKELDQWIEQLNECKQLSESQVKSLCEKAKEILTKESNVQEVRCPVTVCGDVHGQFHDLMELFRIGGKSPDTNYLFMGDYVDRGYYSVETVTLLVALKVRYRERITILRGNHESRQITQVYGFYDECLRKYGNANVW

**Differential Scanning Fluorimetry (DSF)**

To perform the DSF assay, 19 µL of 5 µM protein mixture (PP2A AC dimer or PP2A trimer) were added per well to a clear 96-well PCR plate, 1 µl of SYPRO Orange (100X) was added to make a final 5× SYPRO concentration with a repeat pipettor. The samples were mixed thoroughly, the plate sealed and spun down (500 *g*, 2min). A PCR plate reader was used to visualize fluorescence (25ºC to 95ºC, 0.5ºC/cycle/15 s, total 140 cycles). The measurement was performed in triplicate. Data analysis was performed in Microsoft Excel, the Boltzmann model was used to determine T_m_(1). Curves were plotted in GraphPad Prism.

**In vitro methylation of purified PP2A protein**

Recombinant human active LCMT1 was obtained from Abcam (ab268723). In vitro methylation of PPP2CA was carried out using purified LCMT1 and PP2A protein. First, LCMT1 and PP2A trimer proteins, at a 1:3 molar ratio, was incubated on ice and methylation was initiated by addition of S-adenosyl methionine to a final concentration of 0.75 mM. The reaction was carried out at 22ºC for 4 hours, and the extent of methylation was examined using mass-spectrometry that specifically recognizes the methylated or unmethylated carboxyl terminus of PP2A C-subunit (L309), respectively.

**Phosphatase activity assay**

**Serine/Threonine Phosphatase Assay System V2460 (Promega)** was used to detect phosphatase activity: Briefly, a chemically synthesized phosphopeptide, RRA(pT)VA (PP2A peptide substrate) was diluted in phosphate free water to 0.5 mM. 10 μl of 0.5 mM phosphopeptide, RRA(pT)VA were added per well to a 96-well plate. 40 μl protein phosphatase in 5x colorimetric assay buffer [20 mM Tris-HCl (pH 7.5), 5 mM MgCl_2_, 1 mM EGTA, 0.02% (v/v) betamercaptophenol, 0.1 mg/ml BSA] were added to the same 96-well plate at room temperature (RT). Reaction proceeded for 15 min at RT. Reaction was stopped by adding Molybdate Dye/Additive mixture. Absorbance was read at 620 nm. A no substrate control, no PP2A control, and a phosphate standard were included on each plate. Absorbance was measured in technical triplicate each time and the assay was repeated three times. The presented values are mean values of absorbance, the error bars represent standard deviation.

**Alternatively, p-nitrophenyl phosphate was used to detect phosphatase activity:** Samples of protein phosphatase were diluted in 50 μl in 1× colorimetric assay buffer [20 mM Tris-HCl (pH 7.5), 5 mM MgCl_2_, 1 mM EGTA, 0.02% (v/v) betamercaptophenol, 0.1 mg/ml BSA] in 96-well plate at room temperature (RT). 50 μl of 10 mM p-nitrophenyl phosphate (pNPP) solution was added to each well at regular time intervals (10 s). Reaction proceeded for 20 min at RT. Reaction was stopped by adding 20 μl of 5 M NaOH using the same time interval, Absorbance was read at 405 nm. The molar extinction coefficient for pNPP is 18,000 M^-1^cm^-1^. The blank was subtracted to account for any phosphate release occurring in the absence of phosphatase. Phosphatase activity was measured in triplicate and normalized to the absorbance of the maximum phosphatase activity (determined by using commercial bovine alkaline phosphatase (20 µg/ml final concentration, Sigma). Phosphatase activity was expressed in percentage activity.

**ColabFold Structure Prediction**

Open source ColabFold(2-5) platform was used to determine ternary complex structures. The notebook “ColabFold v1.5.2: Alpha-Fold 2 Multimer using MMseqs2” was accessed via Google Colaboratory platform to run the predictions:

https://colab.research.google.com/github/sokrypton/ColabFold/blob/main/AlphaFold2.ipynb.

The following parameters were used in the notebook – config.json

*{*

*"num_queries": 1,*

*"use_templates": false,*

*"num_relax": 0,*

*"msa_mode": "mmseqs2_uniref_env",*

*"model_type": "alphafold2_multimer_v3",*

*"num_models": 5,*

*"num_recycles": 3,*

*"recycle_early_stop_tolerance": 0.0,*

*"num_ensemble": 1,*

*"model_order": [*

*1,*

*2,*

*3,*

*4,*

*5*

*],*

*"keep_existing_results": false,*

*"rank_by": "multimer",*

*"max_seq": 508,*

*"max_extra_seq": 2048,*

*"pair_mode": "unpaired_paired",*

*"host_url": "https://api.colabfold.com",*

*"stop_at_score": 100.0,*

*"random_seed": 0,*

*"num_seeds": 1,*

*"recompile_padding": 10,*

*"commit": "c7347a34a133fa69db7545d2c9df48fec731074e",*

*"use_dropout": false,*

*"use_cluster_profile": true,*

*"use_fuse": true,*

*"use_bfloat16": true,*

*"version": "1.5.2"*

*}*

Other commits were also present during the runs, but for the same version of the program.

We have used the canonical amino acid sequences of each of the PP2A subunits (A, B and C) for the prediction. The list below summarizes the gene names, UniProt ID’s and amino acid length of the sequence:

A-subunit:

PPP2R1A P30153 (589)

PPP2R1B P30154 (601)

C-subunit:

PPP2CA P67775 (309)

PPP2CB P62714 (309)

B-subunit:

PPP2R2A P63151 (447)

PPP2R2B Q00005 (443)

PPP2R2C Q9Y2T4 (447)

PPP2R2D Q66LE6 (453)

PPP2R5A Q15172 (486)

PPP2R5B Q15173 (497)

PPP2R5C Q13362 (524)

PPP2R5D Q14738 (602)

PPP2R5E Q16537 (467)

PPP2R3A Q06190 (1150)

PPP2R3B Q9Y5P8 (575)

PPP2R3C Q969Q6 (453)

STRN O43815 (780)

STRN3 Q13033 (797)

STRN4 Q9NRL3 (753)

PTPA Q15257 (358)

With the parameters above AlphaFold2Multimer generates 5 prediction models, and for each model computes a per-residue predicted Local Distance Difference Test (pLDDT) value, allowing for assessment of model quality. Another metric to assess the quality of the predicted complex is the Predicted Aligned Error (PAE) of each model which is visualized on a matrix and is used to describe a measure of confidence in position of relative residue pairs. In our analysis we have focused on the highest-ranking (by PAE) model computed by AlphaFold2Multimer. The interface pLDDT were calculated by selecting any amino acids forming inter-chain interactions within 4 Å.

PyMOL command:

*select interface, byres (chain A within 4 of chain B) or (chain B within 4 of chain A) or (chain A within 4 of chain C) or (chain C within 4 of chain A) or (chain B within 4 of chain C) or (chain C within 4 of chain B)*

*average_b interface*

where PyMOL script average_b is defined as described in (https://pymolwiki.org/index.php/Average_b):

*from pymol import cmd,stored*

*def average_b(selection):*

*stored.tempfactor = 0*

*stored.atomnumber = 0*

*cmd.iterate(selection, "stored.tempfactor = stored.tempfactor + b")*

*cmd.iterate(selection, "stored.atomnumber = stored.atomnumber + 1")*

*print("Your selection: %s" % selection)*

*print("sum of B factors: %s" % stored.tempfactor)*

*print("number of atoms: %s" % stored.atomnumber)*

*averagetempfactor = stored.tempfactor / stored.atomnumber*

*print("average B of '%s': %s" % (selection, averagetempfactor))*

*cmd.extend("average_b", average_b)*

**References**

1. Niesen, F. H., Berglund, H., andVedadi, M. (2007) The use of differential scanning fluorimetry to detect ligand interactions that promote protein stability Nat Protoc **2**, 2212-2221 10.1038/nprot.2007.321

2. Mirdita, M., Schutze, K., Moriwaki, Y., Heo, L., Ovchinnikov, S., andSteinegger, M. (2022) ColabFold: making protein folding accessible to all Nat Methods **19**, 679-682 10.1038/s41592-022-01488-1

3. Mirdita, M., Steinegger, M., andSoding, J. (2019) MMseqs2 desktop and local web server app for fast, interactive sequence searches Bioinformatics **35**, 2856-2858 10.1093/bioinformatics/bty1057

4. Mirdita, M., von den Driesch, L., Galiez, C., Martin, M. J., Soding, J., andSteinegger, M. (2017) Uniclust databases of clustered and deeply annotated protein sequences and alignments Nucleic Acids Res **45**, D170-D176 10.1093/nar/gkw1081

5. Mitchell, A. L., Almeida, A., Beracochea, M., Boland, M., Burgin, J., Cochrane, G. *et al.* (2020) MGnify: the microbiome analysis resource in 2020 Nucleic Acids Res **48**, D570-D578 10.1093/nar/gkz1035
